# Raw Cow Milk Consumption and the Atopic March

**DOI:** 10.3389/fped.2021.613906

**Published:** 2021-02-18

**Authors:** Ton Baars, Agnes Wold, Dominique A. Vuitton, Johan Garssen, Anna Catharina Berge

**Affiliations:** ^1^Division of Pharmacology, Utrecht Institute for Pharmaceutical Sciences, Faculty of Science, Utrecht University, Utrecht, Netherlands; ^2^Department of Infectious Diseases, Institute of Biomedicine, Sahlgrenska Academy, University of Gothenburg, Gothenburg, Sweden; ^3^EA 3181, University Bourgogne Franche-Comté, Besançon, France; ^4^Danone Nutricia Research, Utrecht, Netherlands; ^5^Berge Veterinary Consulting BV, Vollezele, Belgium

**Keywords:** cow milk allergy, atopic march prevention, asthma, raw cow milk, risk–benefit

## Introduction

The article “Dietary Prevention of Atopic March in Pediatric Subjects with Cow's Milk Allergy” in Frontiers of Pediatrics, 11 August 2020, reviews potential solutions to the current worldwide increase in cow's milk allergies. The authors state that “In the last years, diet is emerging as a relevant strategy to prevent allergic diseases through the active modulation of among others the immune system.” We agree that there is an urgent need to find new dietary strategies to prevent food allergies as well as the allergic march. However, we feel that the authors of this review miss an important potentially preventive measure, namely raw, unpasteurized farm milk or more gently processed milk.

## Raw Milk in Early Childhood

There are review studies published about the role of raw milk in asthma and allergy (1–3). In more than 90% of epidemiological studies worldwide there was a protective effect of unprocessed cow's milk consumption on the development of asthma, hay fever and atopic sensitization in both in farm and urban children (1). The meta-analysis of Brick et al. (3) including 12 publications, show consistent protective outcomes of early and current raw milk consumption and asthma in both farm and non-farm children. Evidence was especially strong in the ALEX (4) and the PARSIFAL studies (5) in several regions of continental Europe. The study by Perkin and Strachan (6) in Shropshire, UK clearly demonstrated that raw farm milk was equally, or possibly more, effective in children from non-farming households than in farming children. On the other hand, farm children receiving shop milk or boiled farm milk have increased risk of asthma, allergies and atopy in contrast to those drinking raw farm milk (7).

The above studies have been criticized for their cross-sectional methodology, which may have included various types of biases. However, longitudinal observations of the children included in the European case-control cohort “PASTURE,” from 2001 to present, combined with several complementary studies (e.g., the “GABRIELA” studies) supported that consumption of raw milk by the mother and/or the child actually protects against atopic allergy, and this is likely through an early modulation of the immune response (2, 8–10). Epidemiological studies have been evaluated in a meta-analysis and clearly indicate the protective effects of raw milk against asthma and allergies (3).

In addition to the potential tolerance-promoting effect of consumption of raw milk, there are indications that unpasteurized milk may be better tolerated than shop milk by cow's milk allergic children. In a small study eleven children with cow's milk allergy were tested in a double-blind provocation trial with either raw farm milk or pasteurized and homogenized “shop” milk (Table 1). Most children tolerated the maximum amount tested of raw milk (50 ml), but far lower levels of

**Table 1:** Outcomes of pre-clinical studies in allergy and asthma (below) in coherence with a trial in children (above) (abbreviations explained at the bottom of the table).

**Test group human**	**Type of study**	**Milk comparison**	**Outcome symptoms**	**Outcome immunology**	**Publication**
(1) Multiple allergic children, 1½ Y	DBPC Pilot study; Tolerance	Raw, shop	Better tolerance to Raw milk: 50 vs. 8.6 mL of Shop milk	No measurements	Abbring et al. (11) in Clin Exp Allergy. 2019, 00, 1–13; doi: 10.1111/cea.13399
**Test group mice**	**Type of study**	**Milk comparison**	**Outcome symptoms**	**Outcome immunology**	**Publication**
(2) Female C3H/HeOuj mice, 3-5W	Food allergy; Tolerance	Raw, shop	Raw milk: reduced allergic symptoms (skin, shock, temp)	No measurements	Abbring et al. (12) in Nutrients 2019, 11, 1721; doi: 10.3390/nu11081721
(3) Female C3H/HeOuj mice, 3–5W	Food allergy; Tolerance; Epigenetics	Raw, shop	Raw milk: reduced allergic symptoms (skin, shock, temp)	Epigenetic histone modifications	Abbring et al. (12) in Nutrients 2019, 11, 1721; doi: 10.3390/nu11081721
(4) Female C3H/HeOuj mice, 4W	Cow milk allergy; Sensitization	Raw, 80°-heated, Shop	Raw milk: lower allergic potential and less symptoms (skin, shock, temp); higher risk after 80°C compared to 73°C-heated milk	Lower IgE in Raw milkmice; inhibition of Th2 cytokines	Abbring et al. (11) in Clin Exp Allergy. 2019, 00, 1–13; doi: 10.1111/cea.13399
(5) Female C3H/HeOuj mice, 3W	Food allergy; Tolerance	Raw; Steps from 50°C to 80°C-heated milk/30 min	Milk heated at 65°C and higher was no longer protective in terms of skin response	Immunologically active whey proteins started a decrease in concentration at 60°C; Immunoglobulins denaturation at 60/65°C	Abbring et al. (13) in Food & Function, 11, 4982-4993; doi: 10.1039/d0fo01175d
(6) Female C3H/HeOuj mice 3W	Food allergy; Tolerance	Raw, skimmed raw, and pasteurized	Lower allergic potential and less symptoms (skin, shock, temp) after Raw and Skim Raw milk; higher risk after 78°C-heated milk/15 s.	No effects on SCFA in caecum; low IgE and Th2-related cytokines	Abbring et al. (14) in: Nutrients 2019, 11, 1499; doi: 10.3390/nu11071499
(7) Female C3H/HeOuj mice 4W	Cow milk allergy; Sensitization	Native Raw milk whey and 80°C-heated whey	Reduced allergic symptoms (skin, shock, temp) after Raw whey	Reduced Th2 cytokine response in the Raw whey group	Abbring et al. (11) in Clin Exp Allergy. 2019, 00, 1–13; doi: 10.1111/cea.13399
(8) Male BALB/c mice, 6-7W	Asthma: House dust mite allergy	Raw, 80°C-heated Raw	Improved lung function after Raw milk	Reduced number of inflammatory cells	Abbring et al. (15) in Front. Immunol. 8, 1045. doi: 10.3389/fimmu.2017.01045

*DBPC, double blind placebo-controlled trial; skin, ear swelling; shock, anaphylactic shock; temp, reduced body temperature; Th2, T Helper-2 cells; IgE, Immune Globulin E; SCFA, Short Chain Fatty Acids*.

shop milk (8.6 ml on average; *P* < 0.01) (11). In the past 5 years, pre-clinical studies have analyzed various aspects of the raw milk protection against allergic reactions to explain the underlying effects of the protection against the atopic march in children (Table 1: 1).

The differences between raw farm milk and shop milk have been tested in sensitization and tolerance studies in mice (Table 1: 2; 3; 4). When increasing milk heating from 50 to 80°C was tested, it was shown that the allergic responses were present above 60°C in mice (Table 1: 5). Furthermore, in an asthma-mouse model heated raw farm milk (80°C) had a significant impact on the asthma parameters, whereas after raw milk consumption reactions were similar to those observed in the negative control mice (Table 1: 8). These preclinical mice studies indicate that the early life allergenic effects of commercial milk are primarily caused by heating of the heat-sensitive whey protein fraction and not by homogenization or fat standardization (13). This, however, does not preclude other additional factors, like the milk fat composition (n3, n6 FA) and of course more clinical validation is needed.

## Changes in the Raw Milk Matrix

The antigenic properties of raw milk may change after heat treatment. Beta-lactoglobulin (BLG), a protein which is not present in human milk, is considered to be the main allergen in cow's milk. *In vitro* studies showed aggregation and increased antigenicity of BLG when milk is heated above 60°C up to 90°C, while heating above 90°C resulted in a decline of antigenicity, which is known as ‘baked-milk’ (16, 17). Other whey proteins are also reduced in concentration when milk is heated above 60°C (13). Hence, lower pasteurization temperatures and more gentle treatment of milk may be a strategy to increase its tolerability in children with cow's milk allergies.

Further, experimental studies have shown that raw milk ameliorates the allergic reaction. Mice sensitized to house dust mite in an asthma model showed reduced airway responsiveness and eosinophil infiltration when fed raw milk, while heated raw milk did not have this effect (15). Furthermore, raw milk also down-regulated allergic symptoms to an unrelated allergen (ovalbumin) in a food allergy model (13). This implies that heat sensitive whey proteins play a role in tolerance development. Furthermore, the same group of researchers showed that differences in immunogenicity appeared already after heating of the milk to just above 60°C for 30 min, which can be considered a milder treatment than the standard pasteurization procedure. Proteomic studies of heat-treated milk show decreased concentrations of several whey proteins which related to reduced tolerance in the mice model (13).

## Reducing Raw Milk Risks

Naturally, there may be a risk that raw milk contains pathogens that may cause foodborne disease. Thanks to modern technologies and disease eradication and control programs in food producing animals, raw milk production can be carried out safely with closed milking systems and the maintenance of the cold chains. A review of outbreak data in USA associated with raw milk, reported by the Centers of Disease Control, indicates that such outbreaks have been reduced during the last decade, likely due to improved hygiene (18). Certain farmers specializing in producing raw milk for direct consumption may provide microbiologically safe raw milk by employing high hygienic standards. Berge and Baars (19) have provided evidence that various raw milk production systems exist that provide equal microbial safety as pasteurized milk. A sub-study revealed that the raw milk collected in the farms of the PASTURE study participants had an endotoxin content lower than pasteurized and ultra-high temperature processed milk kept in non-farmer family fridges (20). Further, risk—benefit analyses are necessary for raw milk and raw milk products for different types of vulnerable consumers, based on the latest information of safe raw milk production.

## Discussion

Thus, as emphasized by Carrucci et al. (21), the costs for asthma and allergy are increasing. Evidence is accumulating that consumption of raw cow's milk might be an effective preventive strategy that should be further explored. Microbiological safety of such milk must be high priority and strictly controlled. In Western countries this commodity can be produced hygienically and delivered to consumers with existing cold chains to assure that the risk of infectious disease due to its consumption is very low (19). Future research is a must to unravel how safe milk derived products may be produced without damaging important immune modulators. Heating to kill pathogens should not be the only solution to produce microbiologically safe milk. New technological solutions for more gentle processing of milk and training raw milk producers in biosecurity and food hygienic procedures, may minimize the risk of contamination of raw milk by pathogens. In addition, raw milk intended for use in small children may easily be checked for the absence of significant pathogens by rapid PCR procedures before sale of the milk.

## Author Contributions

TB and AB designed and structured the article, wrote, and read the manuscript. All authors listed have made a substantial and intellectual contribution to the work and approved it for publication.

## Conflict of Interest

AB was employed by the company Berge Veterinary Consulting BV, as independent consultants AB and TB perform limited paid consulting on dairy farms and presentations at workshops for raw milk producers. JG is partly employed at Danone Nutricia Research. The remaining authors declare that the research was conducted in the absence of any commercial or financial relationships that could be construed as a potential conflict of interest.
